# Menu-Labeling Usage and Its Association with Diet and Exercise: 2011 BRFSS Sugar Sweetened Beverage and Menu Labeling Module

**DOI:** 10.5888/pcd11.130231

**Published:** 2014-01-02

**Authors:** Kelly M. Bowers, Sumihiro Suzuki

**Affiliations:** Author Affiliations: Kelly M. Bowers, University of North Texas Health Science Center, Fort Worth, Texas.

## Abstract

**Introduction:**

The primary objective of our study was to investigate the association between menu-labeling usage and healthy behaviors pertaining to diet (consumption of fruits, vegetables, sodas, and sugar-sweetened beverages) and exercise.

**Methods:**

Data from the 2011 Behavioral Risk Factor Surveillance System, Sugar Sweetened Beverage and Menu-Labeling module, were used. Logistic regression was used to determine the association between menu-labeling usage and explanatory variables that included fruit, vegetable, soda, and sugar-sweetened beverage consumption as well as exercise.

**Results:**

Nearly half (52%) of the sample indicated that they used menu labeling. People who used menu labeling were more likely to be female (odds ratio [OR], 2.29; 95% confidence interval [CI], 2.04–2.58), overweight (OR, 1.13; 95% CI, 1.00–1.29) or obese (OR, 1.29; 95% CI, 1.12–1.50), obtain adequate weekly aerobic exercise (OR, 1.18; 95% CI, 1.06–1.32), eat fruits (OR, 1.20; 95% CI, 1.12–1.29) and vegetables (OR, 1.12; 95% CI, 1.05–1.20), and drink less soda (OR, 0.76; 95% CI, 0.69–0.83).

**Conclusion:**

Although obese and overweight people were more likely to use menu labeling, they were also adequately exercising, eating more fruits and vegetables, and drinking less soda. Menu labeling is intended to combat the obesity epidemic; however the results indicate an association between menu-labeling usage and certain healthy behaviors. Thus, efforts may be necessary to increase menu-labeling usage among people who are not partaking in such behaviors.

## Introduction

Poor nutrition and obesity are major public health concerns (1,2). In 2011, approximately 69% of adults in the United States were classified as overweight or obese (3,4); if the trend continues, by 2020, an estimated 80% of Americans will be overweight or obese (4). Chronic diseases such as cardiovascular disease, type 2 diabetes, osteoarthritis, and psychological illness are associated with obesity and contribute to increased risk of early death and poor quality of life (5). In addition, these illnesses are associated with billions of dollars in annual health care costs (3). Although a 2006 meta-analytic review of 64 obesity prevention programs and interventions showed that most have been unsuccessful (6), it is important to continue to address the issue because of the growing burden of disease and disability related to obesity.

Obesity is primarily a lifestyle-related condition; it is most often related to a person’s physical activity and nutritional habits. Specifically, it has been linked to consuming few servings of fruits and vegetables and a high level of sweetened beverage consumption (7–12). Although the cause of obesity is complex, frequently dining outside the home is a risk factor for significant weight gain (8,12). Foods prepared in restaurants are generally more caloric and higher in fat, sodium, and sugar than foods prepared in the home (13). The percentage of food expenditures outside the home has increased from 32% in 1980 to 44% in 2010 (14), and more than 50% of Americans eat at fast-food restaurants and other commercially prepared meal establishments approximately 3 times a week (15).

The increase in eating outside the home prompted legislation encouraging healthy eating choices in restaurants. One such legislation model recommends or mandates listing nutritional information next to menu items to increase awareness of the nutritional content of restaurant items and influence healthier food choices (13). Since 2006, several cities and states in the United States have passed laws requiring menu labeling in restaurants. In 2010, as a part of the Patient Protection and Affordable Care Act (PPACA) (16), a law was passed requiring restaurants with 20 or more locations to list caloric information and other nutritional facts about menu items near the point of purchase. Although the implementation of this law has been delayed, many restaurants have already begun displaying calorie and nutritional information.

Although the purpose of displaying such information is clear, the effects are not. Past studies have reported either limited or no differences in nutritional choices when people were provided point-of-purchase menu labeling while dining at a restaurant (8,17). However, it has also been reported that people who used menu labeling to determine calorie content consumed significantly fewer calories during a meal compared with people who did not use menu labeling (13). Additionally, most studies on the effects of menu labeling have been limited to a student population (18) or were restricted to samples from only 1 city (eg, New York, New York [19,20]; Philadelphia, Pennsylvania [21]; Stillwater, Oklahoma [8]). There is a paucity of research on menu labeling on a larger scale. Moreover, little research has been done comparing the differences between people who use menu labeling and those who do not. Many studies considered only the effects that menu labeling have on calorie intake (17,19) while failing to investigate the differences in characteristics between users of menu labeling and nonusers. For this study, we conjectured that users of menu labeling tend to live a healthier lifestyle that includes healthy eating habits and regular exercise while nonusers are people living lifestyles more conducive to becoming overweight or obese. If this were the case, menu labeling (intended to increase awareness of nutritional content and influence healthier food choices) may not help the people who would benefit the most. Thus, the primary objective of our study was to investigate the association between menu-labeling usage and healthy behaviors pertaining to exercise and diet (consumption of fruits, vegetables, sodas, and other sweetened sugary beverages).

## Methods

Data from the 2011 Behavioral Risk Factor Surveillance System (BRFSS) (22) were used. In 2011, the Sugar Sweetened Beverages and Menu Labeling module (Module 4) (23) was administered for the first time, with 3 states, Hawaii, Minnesota, and Wisconsin, implementing the module. The module consisted of 3 questions: the first pertaining to soda consumption, the second to sweetened fruit drinks, and the third to menu-labeling usage at fast-food and chain restaurants. Only participants who responded to questions in Module 4 were included in our study sample. In addition, participants were excluded if they indicated that they “do not eat at fast food or chain restaurants” (n = 1,788), “didn’t know” if they used menu labeling (n = 85), “usually could not find the menu labeling” (n = 97), or refused to answer the question (n = 12). The final sample consisted of 23,951 participants.

### Outcome variable

Menu-labeling usage was dichotomized (use vs nonuse) based on the response to the survey item that read, “The next question is about eating out at fast food and chain restaurants. When calorie information is available in the restaurant, how often does this information help you decide what to order?” Participants who answered either “always,” “most of the time,” “about half the time,” or “sometimes” were collapsed into users (n = 12,587). Participants who answered either “never” or “never noticed or never looked for calorie information” were collapsed into nonusers (n = 11,364).

With no menu-labeling laws enacted in these states at the time of the survey, it is difficult to ascertain what information was available to the subjects. However, because the question in the module specifically asks about calorie information, which is commonly displayed on most menu labels, we assumed that the subjects would be classified as menu-labeling users if they had access to the calorie information.

### Explanatory variables

The primary explanatory variables in regard to diet were consumption of fruits, vegetables, sugar-sweetened beverages, and sodas. Fruit and vegetable consumption information were extracted from BRFSS 2011 Core Section 9: Fruit and Vegetables (24). The information was converted into fruit servings per day and vegetable servings per day. Information on soda and other sugar-sweetened beverage consumption was obtained from Module 4, questions 1 and 2: “About how often do you drink regular soda or pop that contains sugar? Do not include diet soda or diet pop.” and “About how often do you drink sweetened fruit drinks, such as Kool-aid, cranberry, and lemonade? Include fruit drinks you made at home and added sugar to.” The information was converted into soda consumption per day and sweetened fruit beverage consumption per day. In addition, information from BRFSS 2011 Core Section 10: Exercise (24) was used to create the primary explanatory variable for exercise. Whether a participant had attained the recommended aerobic guidelines was measured via a variable provided among the 2011 BRFSS calculated variables, specifically, “_PAINDEX”. This variable indicated whether a subject met daily guidelines for daily aerobic exercise (25). Other covariates included body mass index (BMI), self-perception of general health, age, sex, education level, and annual household income. We did not include race/ethnicity as a covariate because the 3 states used for the analysis did not have enough diversity for a meaningful comparison; however, state was included as a covariate in the full model.

### Statistical analysis

The data were analyzed using SAS 9.3 (SAS Institute Inc, Cary, North Carolina) adjusting for the complex structure of the BRFSS. Univariate analyses were performed using a simple logistic regression model or a χ^2^ test to examine the association between menu labeling usage and various factors. Multivariable logistic regression was used to control for any potential confounders. Statistical tests were determined to be significant for *P* values < .05, but *P* values < .10 were also reported as being marginally significant.

## Results

Nearly half of the sample indicated using menu labeling (52%) (Table 1). Menu-labeling users were more likely to be female (61% vs 39%), have an annual household income of $50,000 or more (49% vs 43%), and exercise according to the aerobic exercise guidelines (60% vs 53%). Similar distribution of BMI categories were found for both users and nonusers. People were more likely to use menu labeling, on average, if they consumed more fruits (OR, 1.43; 95% CI, 1.34–1.54), more vegetables (OR, 1.32; 95% CI, 1.25–1.40), less sugar-sweetened beverages (OR, 0.88; 95% CI, 0.80–0.97), and less sodas (OR, 0.70; 95% CI, 0.64–0.76).

**Table 1:** Descriptive Statistics and Univariate Analysis of Menu-Labeling Users and Nonusers, Behavioral Risk Factor Surveillance System (BRFSS), 2011

Characteristic	Total (n = 23,951)	Users (n = 12,587)	Nonusers (n = 11,364)	Univariate^a^ Odds Ratio (95% CI)
**Self-perception of health, %**				
Fair/poor	13	11	12	1 [Reference]
Good/very good/excellent	87	89	88	1.23 (1.06–1.42)^b^
**Sex, %**				
Male	50	39	61	1 [Reference]
Female	50	61	39	2.44 (2.12–2.69)^b^
**Education, %**				
Less than high school diploma	10	9	10	1 [Reference]
High school diploma or higher	90	91	90	1.26 (1.01–1.57)^b^
**Annual household income, $, %**				
<50,000	53	51	57	1 [Reference]
≥50,000	47	49	43	1.24 (1.12–1.38)^b^
**State, %**				
Wisconsin	45	45	44	1 [Reference]
Hawaii	11	12	11	1.05 (0.91–1.20)
Minnesota	44	43	45	0.89 (0.80–1.01)
**Met aerobic exercise guidelines^c^, %**				
No	42	40	47	1 [Reference]
Yes	58	60	53	1.33 (1.21–1.47)^b^
**Body mass index, kg/m^2^, %**				
Underweight or normal weight	37	38	37	1 [Reference]
Overweight	36	35	37	0.94 (0.84–1.05)
Obese	27	27	26	1.00 (0.88–1.14)
**Continuous measures, mean (standard error)**				
Age, y	46.87 (0.22)	45.91 (0.31)	47.28 (0.31)	0.99 (0.99–0.99)^b^
Soda, daily consumption	0.42 (0.02)	0.30 (0.02)	0.65 (0.05)	0.70 (0.64–0.76)^b^
Sugar-sweetened beverage, daily consumption	0.24 (0.01)	0.21 (0.01)	0.26 (0.02)	0.88 (0.80–0.97)^d^
Vegetable, daily servings	1.52 (0.01)	1.67 (0.02)	1.37 (0.02)	1.32 (1.25–1.40)^b^
Fruit, daily servings	1.03 (0.01)	1.19 (0.02)	0.88 (0.02)	1.43 (1.34–1.54)^b^

Abbreviation: CI, confidence interval.

a *P* values calculated using simple logistic regression.

b *P* < .001.

c As measured via a variable provided among the 2011 BRFSS calculated variables, specifically, “_PAINDEX”. The variable indicated whether or not a subject met daily guidelines for daily aerobic exercise (25).

d *P* < .01.

Multivariable logistic regression was used to further examine the association between menu-labeling usage and the adjusted effects of diet and exercise while controlling for potential confounders (Table 2). The results were mostly consistent with the univariate analyses, which showed that people were more likely to use menu labeling if they met aerobic exercise guidelines (OR, 1.18; 95% CI, 1.06–1.32), consumed more fruits (OR, 1.20; 95% CI, 1.12–1.29), consumed more vegetables (OR, 1.12; 95% CI, 1.05–1.20), and consumed less soda (OR, 0.76; 95% CI, 0.69–0.83). The consumption of sweetened fruit beverages was not significant in the multivariable analysis. The initial multivariable analysis also indicated that, compared with those who are in the underweight or normal weight BMI categories, those who are overweight (OR, 1.13; 95% CI, 1.00–1.29, *P* = .06) and obese (OR, 1.29; 95% CI, 1.12–1.50, *P* < .001) were more likely to use menu labeling.

**Table 2:** Odds Ratios (ORs) of Use of Menu Labeling, Full Multivariable Logistic Regression Model and Models Stratified by Body Mass Index, Behavioral Risk Factor Surveillance System (BRFSS), 2011^a^

Characteristic	Full Multivariable, OR (95% CI)	Underweight or Normal Weight, OR (95% CI)	Overweight, OR (95% CI)	Obese, OR (95% CI)
**Self-perception of health**				
Fair/poor	1 [Reference]	1 [Reference]	1 [Reference]	1 [Reference]
Good/very good/excellent	1.03 (0.86–1.22)	1.15 (0.82–1.62)	0.98 (0.75–1.29)	0.98 (0.75–1.30)
**Sex**				
Male	1 [Reference]	1 [Reference]	1 [Reference]	1 [Reference]
Female	2.29 (2.04–2.58)^b^	1.99 (1.63–2.44)^b^	2.30 (1.92–2.76)^b^	2.44 (2.12–2.69)^b^
**Education level**				
Less than high school diploma	1 [Reference]	1 [Reference]	1 [Reference]	1 [Reference]
High school diploma or higher	1.13 (0.87–1.47)	0.96 (0.62–1.51)	1.40 (0.91–2.17)	1.12 (0.71–1.79)
**Annual household income, $**				
<50,000	1 [Reference]	1 [Reference]	1 [Reference]	1 [Reference]
≥50,000	1.16 (1.04–1.31)^c^	1.25 (1.03–1.51)^d^	1.18 (0.98–1.41)	1.04 (0.83–1.29)
**State**				
Wisconsin	1 [Reference]	1 [Reference]	1 [Reference]	1 [Reference]
Hawaii	1.05 (0.91–1.20)	1.03 (0.92–1.16)	1.23 (0.98–1.53)	1.05 (0.81–1.38)
Minnesota	0.90 (0.80–1.01)	1.20 (1.07–1.34)^c^	1.10 (0.91–1.32)	0.95 (0.76–1.20)
**Met aerobic exercise guidelines^e^**				
No	1 [Reference]	1 [Reference]	1 [Reference]	1 [Reference]
Yes	1.18 (1.06–1.32)^c^	1.14 (0.94–1.41)	1.16 (0.97–1.38)	1.27 (1.03–1.56)^d^
**Body mass index, kg/m^2^**				
Underweight or normal weight	1 [Reference]	NA		
Overweight	1.13 (1.00–1.29)			
Obese	1.29 (1.12–1.50)^b^			
**Continuous measures**				
Age, y	0.99 (0.98–0.99)^b^	0.99 (0.98–0.99)^b^	0.99 (0.98–0.99)^b^	0.99 (0.98–0.99)^b^
Soda, daily consumption	0.76 (0.69–0.83)^b^	0.69 (0.59–0.82)^b^	0.72 (0.64–0.82)^b^	0.84 (0.73–0.96)^d^
Sugar-sweetened beverage, daily consumption	1.00 (0.91–1.10)	0.95 (0.81–1.11)	1.05 (0.89–1.23)	0.99 (0.83–1.19)
Vegetable, daily servings	1.12 (1.05–1.20)^b^	1.03 (0.92–1.16)	1.23 (1.12–1.35)^b^	1.11 (0.99–1.25)
Fruit, daily servings	1.20 (1.12–1.29)^b^	1.20 (1.08–1.34)^b^	1.16 (1.04–1.30)^d^	1.24 (1.08–1.42)^d^

Abbreviation: CI, confidence interval; NA, not applicable.

a *P* values computed using logistic regressions models.

b *P* < .001.

c *P* < .01.

d *P* < .05.

e As measured via a variable provided among the 2011 BRFSS calculated variables, specifically, “_PAINDEX”. The variable indicated whether or not a subject met daily guidelines for daily aerobic exercise (25).

Stratified analyses were conducted for each BMI category to determine why menu-labeling users tend to have healthier dietary and exercise habits than do nonusers of menu labeling yet at the same time tend to be overweight or obese (Table 2). People in the obese category were more likely to use menu labeling if they met aerobic exercise guidelines (OR, 1.27; 95% CI, 1.03–1.56), consume less soda (OR, 0.84; 95% CI, 0.73–0.96), and consume more fruits (OR, 1.24; 95% CI, 1.08–1.42). Vegetable consumption was marginally significant (OR, 1.11; 95% CI, 0.99–1.25, *P* = .09) within the obese category. Similarly, within the overweight category, those who consumed less soda (OR, 0.72; 95% CI, 0.64–0.82) and consumed more fruits (OR, 1.16; 95% CI, 1.04–1.30) tended to used menu labeling more. Meeting aerobic exercise guidelines was marginally significant (OR, 1.16; 95% CI, 0.97–1.38, *P* = .09) within the overweight category.

## Discussion

Menu labeling was used by nearly 50% of the study population; this percentage was considerably higher than past estimates (10%–33%) (19,21,26). This finding may be because these previous studies used a small sample size or a sample restricted to a single city. Although our study includes only 3 states, the sample size is much larger and the sample is representative of the entire state (22). Our results indicated that people were more likely to use menu labeling if they were adequately exercising, eating more fruits and vegetables, and drinking less soda. Past studies demonstrated that menu labeling may contribute to a decrease in calories consumed (8,27). Because we have no measures of actual calorie consumption, we cannot conclude whether menu-labeling usage is the major determinant in decreasing calorie intake or whether people using menu labeling are already participating in healthy behaviors, and hence would naturally consume fewer calories regardless of menu-labeling usage. More research on this matter is needed, because if the former is true, simply increasing menu-labeling usage among the public would lead to intake of fewer calories overall, whereas if the latter is true, then further efforts to increase menu-labeling usage among people who are not participating in such healthy behaviors would be needed.

Harnack and colleagues (28) reported no significant differences with respect to BMI and menu-labeling usage. Although the unadjusted univariate results from our study showed similar findings, the results of the adjusted analysis showed that those who are overweight and obese were more likely to use menu labeling than those who were underweight or normal weight. Hence, at first glance it may seem that menu labeling is being used by the groups most at risk. However, these results are somewhat misleading. Stratified analyses of the data indicate that even among those who are overweight and obese, the people who are using menu-labeling are those participating in healthy behaviors. These people tended to meet aerobic exercise guidelines, consumed more fruits, and consumed less soda than those who did not use menu labeling, suggesting that menu labeling may not be benefiting those who do not partake in such behaviors.

Among the other covariates, menu labeling was more than twice as likely to be used if a person was female. This finding is probably because women are more likely than men to engage in nutrition and exercise activities (29). Women are more likely than men to read the nutrition labels on food items, and when a woman reads nutritional labels, she is more likely to focus on the total amount of calories (30,31). Efforts may be warranted to increase the usage of menu labeling among men.

To our knowledge, this is the first large study on menu labeling; however, several limitations are noted. First, all measures on the BRFSS are self-reported by the participants, which may result in bias. Second, although the BRFSS is structured to provide nationally representative estimates, Module 4 that pertains to menu-labeling usage was administered in only 3 states (Minnesota, Wisconsin, and Hawaii). State was not a significant factor in the model, indicating that combining data from these states was not an issue. However, the results of this study may not be truly representative of the national population. Even so, because Module 4 was implemented for the first time in the 2011 BRFSS, this study may serve as the baseline study for future years when more states implement the module. Third, because of the cross-sectional nature of the BRFSS, it is unknown whether the people using menu labeling were participating in healthy behaviors before they began using menu labeling. Moreover, because no measures of calorie consumption are collected, direct comparisons to other studies with such measures are not possible. Future studies could investigate the lifestyle habits before and after menu-labeling usage while controlling for calorie intake.

Menu labeling is intended to foster a nutritional and health behavior change; however, our study results show an association between menu-labeling usage and participation in healthy behaviors. People using menu labeling reported meeting recommended exercise guidelines, consuming more fruits, and consuming less soda than those who did not use menu labeling. More research is needed to add to the knowledge of menu labeling and its effects on behavior choices.
